# Dose Titration Algorithm Tuning (DTAT) should supersede ‘the’ Maximum Tolerated Dose (MTD) in oncology dose-finding trials

**DOI:** 10.12688/f1000research.10624.3

**Published:** 2017-07-17

**Authors:** David C. Norris

**Affiliations:** 1 Precision Methodologies, LLC, Seattle, USA

**Keywords:** Dose-finding studies, oncology, Phase I clinical trial, individualized dose-finding, precision medicine

## Abstract

**Background**. Absent adaptive, individualized dose-finding in early-phase oncology trials, subsequent ‘confirmatory’ Phase III trials risk suboptimal dosing, with resulting loss of statistical power and reduced probability of technical success for the investigational therapy. While progress has been made toward explicitly adaptive dose-finding and quantitative modeling of dose-response relationships, most such work continues to be organized around a concept of ‘the’ maximum tolerated dose (MTD). The purpose of this paper is to demonstrate concretely how the aim of early-phase trials might be conceived, not as ‘dose-finding’, but as *dose titration algorithm (DTA)*-finding. **Methods. **A Phase I dosing study is simulated, for a notional cytotoxic chemotherapy drug, with neutropenia constituting the critical dose-limiting toxicity. The drug’s population pharmacokinetics and myelosuppression dynamics are simulated using published parameter estimates for docetaxel. The amenability of this model to linearization is explored empirically. The properties of a simple DTA targeting neutrophil nadir of 500 cells/mm ^3^ using a Newton-Raphson heuristic are explored through simulation in 25 simulated study subjects. **Results. **Individual-level myelosuppression dynamics in the simulation model approximately linearize under simple transformations of neutrophil concentration and drug dose. The simulated dose titration exhibits largely satisfactory convergence, with great variance in individualized optimal dosing. Some titration courses exhibit overshooting. **Conclusions. **The large inter-individual variability in simulated optimal dosing underscores the need to replace ‘the’ MTD with an individualized concept of MTD _i_ . To illustrate this principle, the simplest possible DTA capable of realizing such a concept is demonstrated. Qualitative phenomena observed in this demonstration support discussion of the notion of *tuning *such algorithms. Although here illustrated specifically in relation to cytotoxic chemotherapy, the DTAT principle appears similarly applicable to Phase I studies of cancer immunotherapy and molecularly targeted agents.

## Introduction

Despite advances in Bayesian adaptive designs ^1, 2^ and model-based dose-finding ^3^, oncology dose-finding studies remain conceptually in the thrall of ‘the’ maximum tolerated dose (MTD). This fallacious concept stands opposed to the long-recognized heterogeneity of cancer patients’ pharmacokinetics and pharmacodynamics (PK/PD), and to the diversity of their individual values and goals of care. Under this conceptual yoke, these dose-finding studies constitute a significant choke-point in drug development, where a severe discount may be applied to the potential value in new molecules through the hobbling of subsequent ‘efficacy’ trials by inadequate individual-level dosing ^4^.

Strangely, Bayesian innovation in dose-finding studies has proceeded apace without issuing a meaningful challenge to the inherently frequentist conception of an MTD as determined by whole-cohort frequencies of dose-limiting toxicities (DLTs). Thus, even as Bayesianism has made progress toward the ethical imperative of *efficient use of data* ^5^ in such studies, it has neglected to confront the distinct ethical dimension of *individualism* ^6^. This seems a great irony, as the dynamic learning model of Bayesianism is equally suited, and indeed equally essential, to solving the latter problem.

This paper demonstrates individualized dose-finding in a simulated Phase I study of a cytotoxic chemotherapy drug for which neutropenia constitutes the critical dose-limiting toxicity. Importantly, myelosuppression is interpreted also as a monotone index of therapeutic efficacy, without the added complication of a dose-response ‘plateau’ ^7^ such as postulated for molecularly targeted agents (MTAs). This creates a problem setting where simple heuristics apply, simplifying the demonstration undertaken here. The aim of this exercise is to elaborate a concrete setting in which ‘dose-finding study’ may readily be seen as a misnomer. Under the view advanced here, early-phase studies of this kind should be conceived as *dose titration algorithm tuning* (DTAT) studies.

The idea that ‘dose finding studies’ should yield dose titration algorithms (DTAs) is not new. More than a quarter-century ago, Sheiner and colleagues ^8^ advocated a learn-as-you-go concept for “dose-ranging studies”, addressing concerns about “parallel-dose designs” that are not far removed from the motivations for the present paper. As in the advocacy of Sheiner *et al*., parametric models play an important role in this paper, although in keeping with a spirit of pragmatism I relax this dependence to some extent by means of a semiparametric dynamic on-line learning heuristic.

## Methods

A hypothetical cytotoxic drug is considered, modeled notionally after docetaxel, to be infused in multiple 3-week cycles. The pharmacokinetics are taken to follow a 2-compartment model with parameters as estimated for docetaxel in a recent population pharmacokinetic study ^9^. Chemotherapy-induced neutropenia (CIN) is taken to follow a myelosuppression model due to Friberg *et al.* ^10^. Together, these models form a population pharmacokinetic/pharmacodynamic (PK/PD) model within which DTAs may be simulated and tuned for optimality. For simulation purposes, and anticipating the future value of ready access to a variety of inference procedures in follow-on work, this PK/PD model is implemented in R package **pomp** ^11^. R version 3.3.2 was used ^12^.

Basic behaviors of the models are illustrated by simulation graphics generated for 25 individuals randomly generated from the population PK/PD model. Properties with specific relevance to absolute neutrophil count nadir ( *ANC _nadir_*)-targeted dose titration are then investigated, with an eye to demonstrating the predictability of nadir timing. In particular, an approximate linearization of neutrophil nadir level and timing is demonstrated, achieved through suitable power-law transformation of infusion doses and logarithmic transformation of neutrophil concentration. Within this transformed parameter space, a simple recursive DTA is defined on the basic heuristic of the Newton-Raphson method for root-finding. For simplicity, monitoring of CIN is not modeled endogenously to this algorithm, but is treated as exogenous such that nadir timing and level are known precisely. A ‘DTAT’ study is simulated and visualized for 25 patients, with the *tuning parameters* of the recursive titration algorithm held fixed. The visualization supports a discussion of how these parameters might be *tuned* over the course of a Phase I study. All simulations and figures in this paper were generated by a single R script, archived on OSF ^13^.

### Pharmacokinetic model

We take the population pharmacokinetics of our cytotoxic drug to obey a 2-compartment model, with parameters drawn notionally from estimates published for docetaxel [ 9, Table 2]; see Table 1.

**Table 1.** Two-compartment pharmacokinetics of docetaxel, from Onoue *et al.* (2016) [ 9, Table 2]. *CL*: clearance; *Q*: intercompartmental clearance; *V _c_*: volume of central compartment; *V _p_* volume of peripheral compartment; *CV*: coefficient of variation. (*) A CV for *V _c_* was unavailable in 9 and has been set arbitrarily to 0.1.

Param	Units	Mean	CV
*CL*	L/hour	32.6	0.295
*Q*	L/hour	5.34	0.551
*V _c_*	L	5.77	0.1*
*V _p_*	L	11.0	0.598

**Figure 1.**
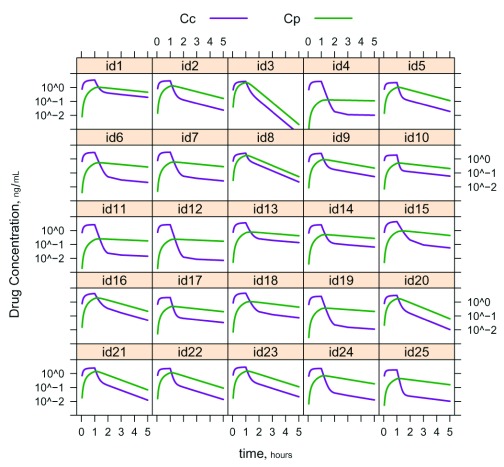
Two-compartment pharmacokinetics of a 1-hour infusion of 100 mg of the modeled drug, for 25 randomly generated individuals in our population pharmacokinetic model. *C _c_* and *C _p_* are drug concentrations in the central and peripheral compartments, respectively.

Figure 1 shows illustrative pharmacokinetic profiles for 25 randomly-generated individuals from this population, administered a 100 mg dose.

### Myelosuppression model

**Figure 2.**
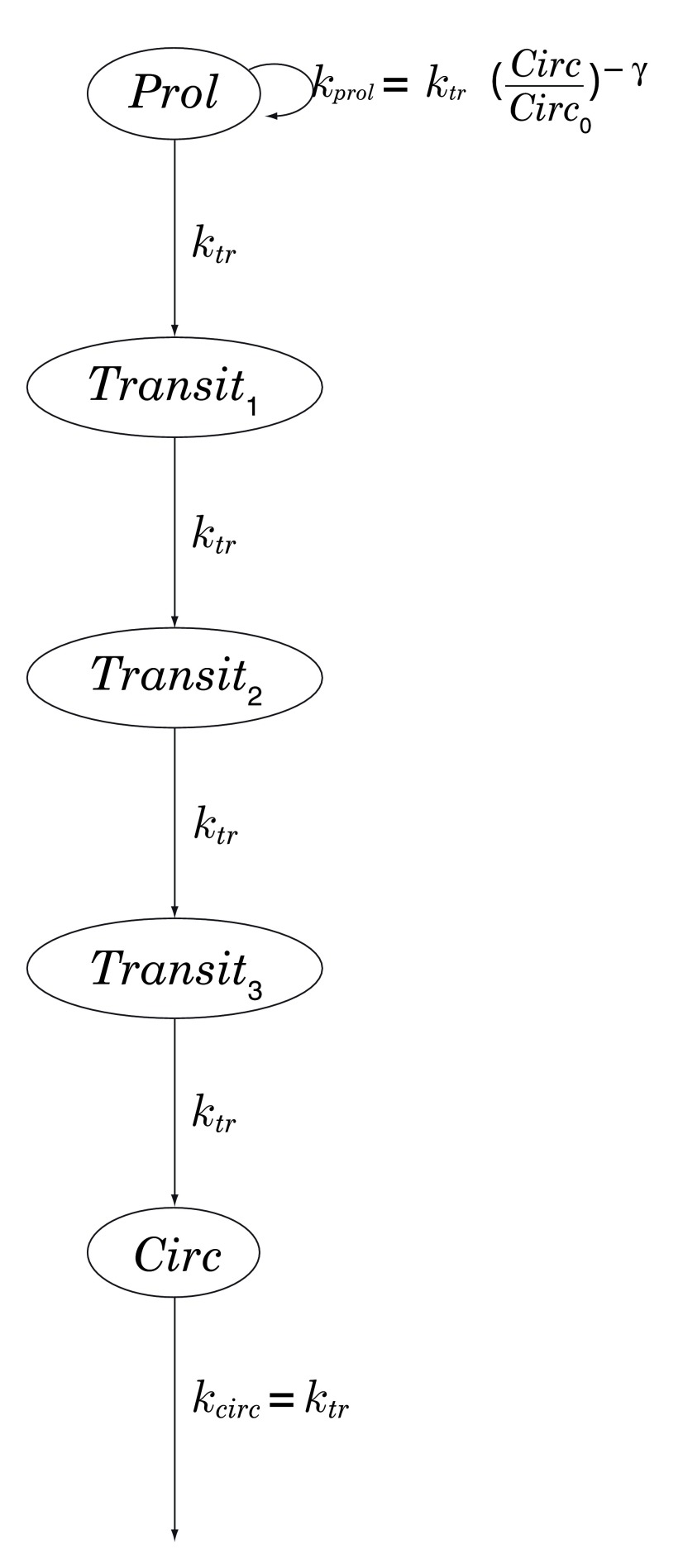
Chemotherapy-induced myelosuppression model of Friberg *et al.* [ 10, Table 4]. *Prol*: proliferative compartment; *Transit _n_*: maturation compartments; *Circ*: systemic circulation; *k _tr_*: transition rate; *k _prol_*: rate of proliferation of progenitor cells, regulated by a negative-feedback loop parametrized by *γ* > 0.

Chemotherapy-induced neutropenia is simulated using the 5-compartment model of Friberg *et al.* [ 10, Table 4], in which myelocytes (here, neutrophils) arise from progenitor cells in a proliferative compartment, mature through a series of 3 transitional states, and emerge into the systemic circulation; see Figure 2. Transit between successive compartments in this model is a Poisson process with time constant *k _tr_*, total mean transit time being therefore given by *MTT* = 4 */k _tr_*. See Table 2.

**Table 2.** Parameters of the chemotherapy-induced myelosuppression model of Friberg *et al.* [ 10, Table 4]. *Circ*_0_: baseline neutrophil concentration; *MTT* : mean transit time between the 5 model compartments; *γ*: exponent of feedback loop; *EC*_50_, *E _max_*: parameters of a model (of the standard E _max_ type) governing docetaxel-induced depletion in the proliferative compartment.

Param	Units	Mean	CV
*Circ*_0_	cells/mm ^3^	5050	0.42
*MTT*	hours	89.3	0.16
*γ*	-	0.163	0.039
*E _max_*	*µ*M	83.9	0.33
*EC*_50_	*µ*M	7.17	0.50

**Figure 3.**
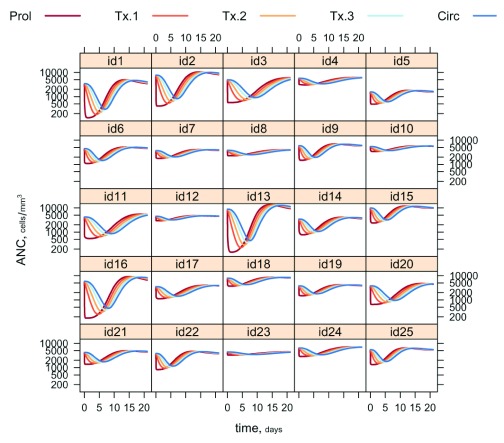
Myelosuppression profiles for the same 25 randomly generated individuals as in Figure 1. Note how a chemotherapeutic ‘shock’ to the proliferative compartment *Prol* propagates through the maturation compartments *Tx*_1,2,3_ and thence to the systemic circulation *Circ*. ( *ANC*: absolute neutrophil count.)

Figure 3 shows illustrative myelosuppression profiles for 25 randomly-generated individuals from this population, administered a 100 mg dose.

### Linearizing CIN dynamics by dose rescaling

**Figure 4.**
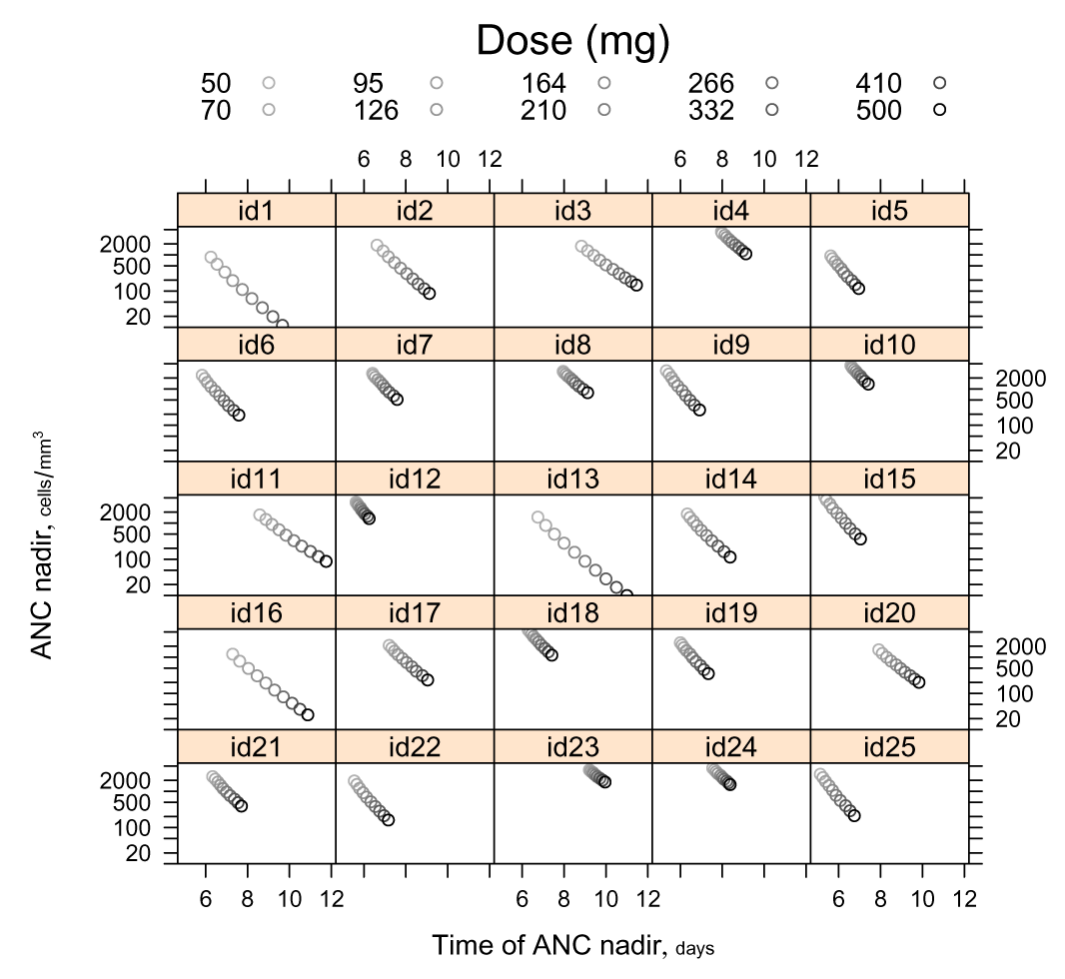
Trajectories of ANC nadirs during dose escalation in 25 randomly-generated individuals. The 10 doses plotted are evenly spaced on a fourth-root scale. Not only are the trajectories themselves nearly linear in (log( *ANC*) × *time*)-space, but each one is traversed at roughly ‘constant velocity’ with respect to $\sqrt[{4}]{dose}$.

When parametrized by $\sqrt[{4}]{dose}$, individuals’ trajectories in (log( *ANC _nadir_*) *×time _nadir_*)-space may be approximately linearized, as shown in Figure 4. This linearization recommends the fourth-root and logarithmic transformations employed hereafter for drug dose and ANC.

## Dose titration

Recursive nonlinear filtering, as implemented in the extended Kalman filter (EKF) or its more modern adaptations ^14^, constitutes a powerful conceptual framework for approaching model-based dose titration ^15^. Indeed, the ‘tuning’ in ‘DTAT’ was itself suggested by the practice of tuning a Kalman filter ^16^ for optimal performance.

For present purposes, however, it suffices to implement a model-free recursive titration algorithm built on the Newton-Raphson method, with a numerically-estimated derivative based on most recent infusion doses and their corresponding ANC nadirs. In this algorithm, a relaxation factor *ω* = 0.75 is applied to any proposed dose *increase*, with safety in mind. Whereas the slope of log( *ANC _nadir_*) with respect to $\sqrt[{4}]{dose}$ is expected to be strictly negative *at steady state*, hysteresis effects arising during initial steps of dose titration do sometimes yield positive numerical estimates for this slope; so the slope estimates are constrained to be ≤ 0. The infusion dose for cycle 1 is 50 mg, and the cycle-2 dose is calculated conservatively using a slope *−*2.0, which is larger (in absolute terms) than for any of our simulated patients except id1 and id13; see Figure 4. For reference, these starting values for the *tuning parameters* of the titration algorithm are collected in Table 3.

**Table 3.** Values of the *tuning parameters* of the dose titration algorithm simulated in Figure 5.

Param	Description	Value
*ω*	Relaxation factor	0.75
*slope*_1_	Slope for cycle-2 dosing	*−*2.0
*dose*_1_	Initial (cycle-1) dose	50 mg

With the illustrative purpose of this article again in mind, we treat *neutropenia monitoring* as an exogenous process yielding precise nadir timing and levels. This enables a demonstration of the main point without the encumbrance of additional modeling infrastructure peripheral to the main point.

## On ‘tuning’

**Figure 5.**
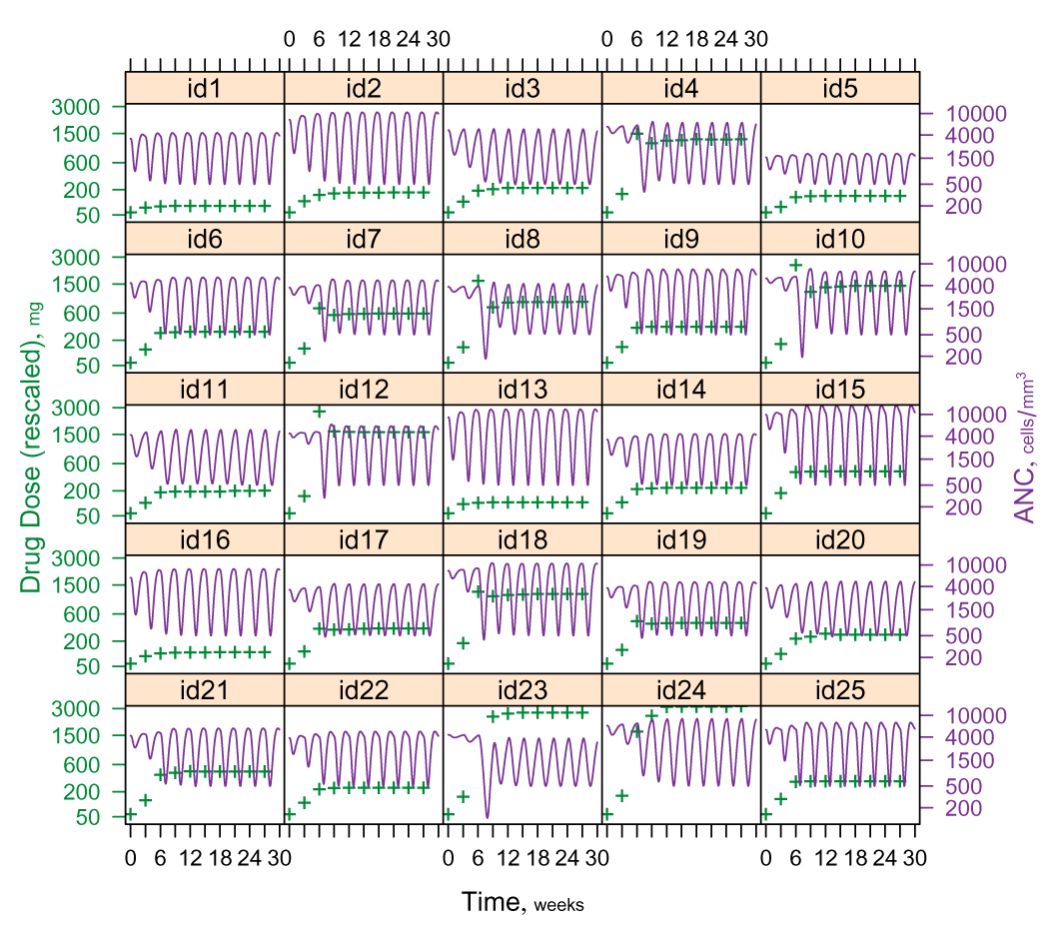
Titration profiles in 25 simulated patients over ten 3-week cycles of chemotherapy. Note particularly the overshooting that occurs in subjects id8, id10, id12 and id23. This underscores the importance, in actual DTAT applications, of imposing fail-safe upper bounds both on *absolute dose* and on *relative dose* increases. It also bears noting that the 25 MTD _i_’s evident in this figure span 1.5 orders of magnitude.

If one considers Figure 5 as a *sequence* of titration outcomes emerging in serially enrolled study subjects, it becomes clear that even quite early in the study it will seem desirable to ‘retune’ the titration algorithm. For example, provided that course-1 CIN monitoring is implemented with sufficient intensity to deliver advance warning of an impending severely neutropenic nadir, so that timely colony-stimulating factor may be administered prophylactically ^17^, then upon review of the titration courses in the first 10 subjects it may well appear desirable to increase *dose*_1_ from 50mg to 100mg. Likewise, given the third-dose overshooting that occurs in 4 of the first 10 subjects, it may seem desirable to adjust the relaxation factor *ω* downward. Of note, at any given time any such proposed retuning may readily be subjected to a ‘dry run’ using retrospective data from all convergent titration courses theretofore collected. (Hysteresis effects would however be inaccessible to a strictly data-driven dry run absent formal modeling that captures such effects.) Furthermore, the ‘tuning’ idea readily generalizes to the fundamental modification or even wholesale replacement of a DTA. For example, the overshooting seen for subjects id8, id10, id12 and id23, inspires further thought about refining (or replacing) the admittedly very naive Newton-Raphson method employed herein. (At the very least, DTAs deployed in actual DTAT studies must incorporate fail-safe upper bounds both on *absolute dose* and on *proportional dose* increases.)

A further dimension of ‘tuning’ that must be discussed is the potential for driving the tuning parameters using statistical models built on baseline covariates. Surely, to the extent that the great heterogeneity in final dosing evident in Figure 5 could be predicted based on *age*, *sex*, *weight* or indeed on pharmacogenomic testing, then *dose*_1_ should be made a function of these covariates. The *recalibration* of such models as data accumulate from successive study subjects is very much a part of the full concept of ‘tuning’ I wish to advance.

Finally, whereas I have discussed ‘tuning’ here largely in terms of reflective, organic decision-making such as occurs in the creative refinement of algorithms or in data-driven statistical model development, I do not mean to exclude more formal approaches to algorithm tuning. A decision-theoretic framing of the tuning problem should enable formal algorithm tuning to be specified and carried out meaningfully. Such framing would also have the salutary effect of bringing into view *objectively* the important matter of patients’ heterogeneity with respect to values and goals of care. It seems quite likely that the balance of benefits from *aggressive titration* versus harms of *toxicities* will generally differ from one patient to another. Dose titration algorithms should most emphatically be tuned to these factors as well. For example, if a patient with more advanced disease and short expected survival nevertheless decided to enroll in a Phase I DTAT study to pursue the possibility of therapeutic benefit, then this patient’s decision would indicate a subjective weighting of benefits vs. harms favoring a *higher starting dose* and *more aggressive titration*.

## Discussion

It is where pharmacometrics meets the field of *optimal control* that the current literature seems to make its closest point of contact with the DTAT concept I am advancing here. In optimal-control investigations of chemotherapy ^18– 23^, as in DTAT, relatively large decision spaces are explored. Indeed, the infinite-dimensional spaces of *control functions* posited for exploration in optimal control applications dwarf the finite-dimensional spaces of *tuning parameters* in DTAT as dramatically as the latter dwarf the finite sets of *discrete doses* trialed in now-standard Phase I studies. This intermediate ‘cardinality’ of DTAT reflects an important advantage in an era when, to almost universal chagrin, the detested 3+3 dose-finding design retains its hegemony due partly to widespread resistance to modeling ^24^. In such an era, optimal control applications that involve detailed mathematical modeling of *tumor biology and dynamics* sadly seem consigned to the fringes of practice. Acceptance of such ambitious problem formulations, expressing as they do the spirit of a future age, must await deep cultural changes in the medical sciences and clinical practice.

As easy as it is, however, to disparage ‘resistance to modeling’ as some kind of antediluvian attitude, this resistance does rightfully assert the importance of unmodeled complexities that necessitate application of organic forms of clinical judgment ^25^. It should be clear from the above discussion of ‘tuning’ that DTAT readily accommodates and veritably *invites* scrutiny, supervision and modification by clinical judgment. For example, if during the course of our DTAT study adverse effects other than neutropenia were to emerge as occasional dose-limiting toxicities, then the full concept of ‘tuning’ advanced above would invite dynamic, ‘learn-as-you-go’ modifications of the titration algorithm. Such modifications could begin with decreasing the relaxation factor *ω*, but might also involve efforts to classify and predict these new DLTs, and to incorporate such new understanding explicitly into the DTA yielded by the study. Indeed, whatever philosophical challenge DTAT embodies is likely to take the form of requiring an *intensified* commitment to clinical judgment, in a learn-as-you-go world where the always-provisional nature of medical knowledge must frankly be acknowledged ^6, 26^.

I have presented the *DTAT principle* here embedded in the context of a specific simulation study. This creates the need explicitly to demarcate what I wish to advance as essential in DTAT, from what is merely incidental to the illustration offered here. DTAT makes its fundamental contribution in putting forward a **new abstraction** (the *DTA* with its *tuning parameters*) capable of *embodying knowledge objectively* ^27^, to supersede a **fallacious abstraction** (‘the’ MTD) that almost completely lacks this capability. I use the term *fallacious* advisedly, meaning to identify *‘the’ MTD* specifically with what Whitehead called the “ *fallacy of misplaced concreteness* [which] consists in neglecting the degree of abstraction involved when an actual entity is considered merely so far as it exemplifies certain categories of thought ^28^.” Indeed, the 1.5 orders of magnitude spanned by the MTD _i_’s of Figure 5 show the degree of abstraction involved in *‘the’ MTD* to be so egregious as to render this concept plainly useless for embodying what we need to learn from Phase I oncology studies.

Several aspects of the illustration offered here should be clearly understood as *not* essential to the DTAT principle. Firstly, notwithstanding the important heuristic role it has played in the development (and even the naming) of DTAT, *recursive filtering* in no way delimits DTAT. In fact, I now rather suspect that full-information methods will push recursive filtering to the sidelines in practical DTAT applications, and that whatever utility recursive filtering retains will derive from its use as a vehicle for illustrating DTAT to clinicians, perhaps in nomogram forms ^15^. Secondly, although neutropenia-targeted dosing of a cytotoxic chemotherapy drug has provided a most propitious context for the present simulation, DTAT need not be thought limited to such drugs. In the important area of immuno-oncology, common dose-limiting toxicities (DLTs) admit monitoring on time-scales comparable to the chemotherapy induced neutropenia (CIN) simulated here. For example, the *cytokine release syndrome* (CRS) that accompanies chimeric antigen receptor (CAR-)T cell therapies typically arises within 1 week of administration (even earlier with concomitant high-dose IL-2) and constitutes a clinical *syndrome* that admits multivariate monitoring on numerous quantitative clinical and laboratory measures ^29^. Even molecularly targeted agents (MTAs), for which *late* toxicities have attracted the lion’s share of attention ^30^, remain accessible to the DTAT principle—with DTA learning occurring on a longer time scale. Of course, a DTA that reacts to diverse, lower-grade MTA toxicities ^31^ that patients *experience and evaluate subjectively* may resemble a process of ongoing *shared decision making* (with the oncology care team) more closely than it resembles the impersonal calculations we typically think of as ‘algorithmic’. But with a suitably broadened understanding of ‘algorithm‘—one that accommodates what might typically be termed *protocols*—the DTAT (or perhaps, DT *P*T) principle continues to apply. In such applications, *supervision and modification by clinical judgment* as mentioned above clearly comes to the fore. But even then, the development and application of scoring systems for patient-reported clinical symptoms and quality of life would enable dose titration protocols (DT *P*s) to be described objectively in quite ‘algorithmic’ terms that would preserve the applicability of a ‘tuning’ concept.

## Conclusions

I have advanced a concept of *dose titration algorithm tuning* (DTAT), drawing illustrative and orienting connections with *recursive filtering* and *optimal control*. I have concretely illustrated key elements of DTAT by simulating neutrophil-nadir-targeted titration of a hypothetical cytotoxic chemotherapy drug with pharmacokinetics and myelosuppressive dynamics patterned on previously estimated population models for docetaxel. I have also discussed the applicability of DTAT to other types of anti-cancer therapy. I believe DTAT presents a *prima facie* case for discarding the outmoded concept of ‘the’ maximum tolerated dose (MTD) of cancer therapeutics. This argument should be of interest to a wide range of stakeholders, from cancer patients with a stake in receiving optimal individualized ‘MTD _*i*_’ dosing, to shareholders in pharmaceutical innovation with a stake in efficient dose-finding before Phase III trials.

## Data availability

The data referenced by this article are under copyright with the following copyright statement: Copyright: © 2017 Norris DC

*Open Science Framework:* Code and Figures for v1 of F1000Research submission: Dose Titration Algorithm Tuning (DTAT) should supersede the Maximum Tolerated Dose (MTD) concept in oncology dose-finding trials, doi 10.17605/osf.io/vwnqz ^13^

## Endorsement

Daniela Conrado (Associate Director, Quantitative Medicine at Critical Path Institute) confirms that the author has an appropriate level of expertise to conduct this research, and confirms that the submission is of an acceptable scientific standard. Daniela Conrado declares she has no competing interests.
