# Supplementary material for: Dose Titration Algorithm Tuning (DTAT) should supersede ‘the’ Maximum Tolerated Dose (MTD) in oncology dose-finding trials
Source: F1000Res. 2017 Jul 17;6:112. Originally published 2017 Feb 7. [Version 3] doi: 10.12688/f1000research.10624.3 (PMC5473410; doi:10.12688/f1000research.10624.3)
Supplement: Peer review history

# Peer review: Referee response for version 2

**DOI:** 10.5256/f1000research.12185.r23469

**Published:** 2017-06-14

**Authors:** Yuan Ji

**Affiliations:** 1 Department of Public Health Sciences, University of Chicago, Chicago, IL, USA

I agree fully that MTD should not be the only goal of dose finding. The paper nicely pointed that out. The author seems to suggest using an optimization approach (i.e., Newton-Raphson) rather than a model-based approach for achieving individualized dosing. There is no concrete design being proposed, or at least it is unclear to me how to design a trial based on the contents in the paper.

 Also, I think the paper can be more concise and needs some more clear flow on the statistics and mathematical modelling. It is expected that a probability model (or an algorithm) is presented with an inference procedure. There are a few models proposed in this paper, but their relationship to the inference is a bit unclear. I would recommend a subsection of “Algorithm” that would connect different parts of the paper into a coherence piece. Otherwise, the paper clearly discusses an important issue in dose finding.

I have read this submission. I believe that I have an appropriate level of expertise to confirm that it is of an acceptable scientific standard.

# Response

**Published:** 2017-06-21

**Authors:** David C. Norris

**Affiliations:** Precision Methodologies, LLC, USA

I thank Dr. Ji for his thoughtful and encouraging review. I acknowledge that this paper by itself does not provide a recipe for designing a complete clinical trial. My aim here has been rather to offer a philosophical and conceptual framework within which such designs may be conceived and constructed. Thus, as the saying goes, "Some User Assembly Required." **I do wish to encourage ambitious research teams in academia and industry to take up this challenge.** I trust that its formidable nature will attract, rather than deter, the necessary sort of interest.

A great deal more will be required to design a DTAT study than has hitherto been required to realize current designs organized around 'the' MTD. By its nature, 'the' MTD tends to abstract away all details of PK/PD, and thus to enable biostatistician trialists to proceed unimpeded by entanglements with pharmacometrics. 'The' MTD also lends itself rather easily to the construction of simple probability models, and to statistical inference over a scant few parameters. No doubt, 'the' MTD owes its astonishing longevity (despite its *prima facie* invalidity) in part to its having enabled such functional compartmentalization and easy statistical modeling. Research groups that successfully develop meaningful DTAT designs will surely comprise interdisciplinary collaborations spanning *pharmacometrics*, *biostatistics* and the *clinical* realm. Such collaborations, although perhaps difficult (Kowalski 2015, Senn 2010, Sheiner 1991), are ripe with riches for those who will pull them off ( Norris 2017c).

From the perspective of *Biostatistics*, a formal probability model and associated statistical inference over its parameters are conspicuously absent from this paper. While I anticipate that formal statistical inference will usefully illuminate the DTAT principle and its applications, I doubt that it is strictly necessary to them. Formal inference in a DTAT application will likely find itself subordinated to "organic forms of clinical judgement" such as I reference in the Discussion section. Indeed, any "dynamic, 'learn-as-you-go' modifications" of a DTA would effectively pull the rug out from under a statistical inference paradigm that requires *ex ante* commitments to immutable probability models. Thus, statistical model development might potentially extend *into and throughout* the conduct of a DTAT study, so as to give a DTAT study partly the character of what is commonly called 'data-driven model development'. Performing statistical inference over the tuning parameters of a DTA intrinsically has a quite different character and level of difficulty from inference over the probability of a DLT (Rogatko *et al* 2015). The most meaningful applications of the DTAT principle will employ models incorporating diverse mechanistic biological insights informed by disparate sorts of data (Senn 2010). Not all such 'informing' will be feasibly accomplished entirely under the duress of statistical formality.

Nevertheless, I do hope in forthcoming publications to demonstrate explicitly how formal statistical inference may play a useful role in a DTAT study; as I mention in the Methods section, I have selected the **pomp** package for implementing this early work precisely to avail myself of a flexible platform for later incorporating a variety of inference procedures. **I heartily welcome expressed interest from potential collaborators in industry, academia and the regulatory sphere.**

**References**

Kowalski KG. My Career as a Pharmacometrician and Commentary on the Overlap Between Statistics and Pharmacometrics in Drug Development. *Statistics in Biopharmaceutical Research*. 2015;7(2):148-159. doi: 10.1080/19466315.2015.1008645.

Senn S. Statisticians and pharmacokineticists: what they can still learn from each other. *Clin Pharmacol Ther*. 2010;88(3):328-334. doi: 10.1038/clpt.2010.128.

Sheiner LB. The intellectual health of clinical drug evaluation. *Clin Pharmacol Ther*. 1991;50(1):4-9.

Norris DC. Costing 'the' MTD. *bioRxiv*. June 2017. doi: 10.1101/150821.

Rogatko A, Cook-Wiens G, Tighiouart M, Piantadosi S. Escalation with Overdose Control is More Efficient and Safer than Accelerated Titration for Dose Finding. *Entropy (Basel)*. 2015;17(8):5288-5303. doi: 10.3390/e17085288.

# Peer review: Referee response for version 2

**DOI:** 10.5256/f1000research.12185.r22676

**Published:** 2017-05-18

**Authors:** Felipe Maglietti

**Affiliations:** 1 Faculty of Exact and Natural Sciences, University of Buenos Aires, Buenos Aires, Argentina

It is a very interesting article. We should start moving forward to a more personalized medicine. Dosing, specially in oncology is very complex since in many cases the short life expectancy can be ruined by having serious side effects. This approach could be very useful for determining the optimal dose in different patients.

I have read this submission. I believe that I have an appropriate level of expertise to confirm that it is of an acceptable scientific standard.

# Peer review: Referee response for version 1

**DOI:** 10.5256/f1000research.11448.r21177

**Published:** 2017-03-22

**Authors:** Natalja Strelkowa

**Affiliations:** 1 Global Biometry and Clinical Applications, Boehringer Ingelheim Pharma GmbH & Co. KG, Biberach an der Riss, Germany

I support the main idea of the article to promote individualized dose finding for toxic drugs. The methodological framework of reinforcement learning is an interesting and promising alternative to the current practice of population based maximum tolerated dose approach (MTD).

 The following points need to be stressed and/or addressed in the paper:

- The immediate toxic response must be reliably measurable for the reinforcement learning to work; long term toxicity is as far as I have understood is not included in the current framework. This needs to be addressed, in particular in light of new immune-oncology and targeted therapies where the toxic response is not immediately observable.
- The time to achieve optimal dosing scheme according to the dose finding algorithm is also of a key importance and must be much shorter than expected survival of the patient.
- For the practical implementation of the approach, hart boundaries on overshooting must be implemented. This will likely influence the time to optimal dosing for each patient.

The title and the conclusions are too strong in my view and need revision. This approach is very promising and I myself am also very interested in its application, but in the abstract and discussion section it needs to be clear that key practical questions are still to be addressed. Strong statements like: ‘DTAT should supersede MTD’ should be removed from the title and abstract and replaced by ‘DTAT is an alternative to current practice’ or ‘Could be a better alternative to current practice for drugs with narrow therapeutic window’ etc.

I have read this submission. I believe that I have an appropriate level of expertise to confirm that it is of an acceptable scientific standard, however I have significant reservations, as outlined above.

# Response

**Published:** 2017-03-23

**Authors:** David C. Norris

**Affiliations:** Precision Methodologies, LLC, USA

I thank Dr. Strelkowa greatly for her supportive and critical comments, which invite extended discussion on several points that v1 of this paper has omitted **to its detriment**. I use this reply to indicate changes I propose to make in v2, pursuant to Dr. Strelkowa’s comments. Unless otherwise indicated, these changes would seem to belong in the **Discussion** section:

- Dr. Strelkowa rightly points to general conditions that constrain the time-scale on which DTAT-based learning may operate. Specifically, DTAT cannot learn faster than the lag-time at which the targeted toxic response(s) develop. In the important area of immuno-oncology that Dr. Strelkowa highlights, common dose-limiting toxicities (DLTs) do admit monitoring on time scales comparable to the chemotherapy induced neutropenia (CIN) simulated in this paper. For example, the *cytokine release syndrome* (CRS) that accompanies chimeric antigen receptor (CAR-)T cell therapies typically arises within 1 week of administration (even earlier with concomitant high-dose IL-2) and constitutes a clinical *syndrome* that admits multivariate monitoring on numerous quantitative clinical and laboratory measures.[1] Regarding however the molecularly targeted agents (MTAs) also mentioned by Dr. Strelkowa, *late* toxicities indeed have tended to attract the lion’s share of attention.[2] One reason for this is that the early toxicities of MTAs tend to be relatively milder than those of cytotoxic and immunologic therapies.[3] Nevertheless, a DTAT principle continues to apply here—just on a longer time scale. Of course, a ‘dose titration *algorithm’* (DTA) that responds to MTA toxicities that patients mainly *experience and evaluate subjectively* may resemble a process of ongoing *shared decision making* (with the oncology care team) more than it resembles the impersonal calculations we typically think of as ‘algorithmic’. But with a suitably broadened understanding of ‘algorithm’—one that accommodates what might typically be termed **protocols**—the DTAT (or perhaps, DT **P**T) principle continues to apply. In such applications, “supervision and modification by clinical judgment” as mentioned in the **Discussion** clearly comes to the fore. But even in such contexts, the development and application of scoring systems for patient-reported clinical symptoms and quality of life would enable dose titration **p**rotocols (DT **P**s) to be described objectively in quite ‘algorithmic’ terms that would preserve the applicability of a ‘tuning’ concept.
- The issue of *competing risks* Dr. Strelkowa raises is truly important, and brings into play the inter-individual heterogeneity in “values and goals of care” that I discussed in opening the **Introduction** and (especially) toward the end of the **On ‘tuning’** section, where I explicitly highlight values/goals as factors that should “most emphatically” inform DTA tuning. (In the PDF, this utterly essential point tragically breaks across pages 7-9 due to an intervening page 8 of figures, a mishap I will aim to avoid in v2.) To address Dr. Strelkowa's comment here, I plan to expand that latter discussion explicitly to incorporate *prognosis*. For example, if a patient with more advanced disease and short expected survival has (in consultation with the oncologist and oncology care team) nevertheless decided to enroll in a Phase I DTAT study to pursue the possibility of therapeutic benefit, then this patient’s decision indicates a subjective weighting of benefits vs harms favoring a *higher starting dose* and *more aggressive titration*.
- I agree that any sound dose titration algorithm will incorporate fail-safe limits on dose escalation. To support a richer discussion in the **On ‘tuning’** section, I purposely chose ‘wrong’ tuning parameters (and a naive Newton-Raphson DTA) so that Figure 5 would illustrate several problems that one would never purposely ‘design in’ to an actual study: (a) a too-low starting dose and (b) the potential for overshooting, such as occurred in id8, id10, id12 and id23—the last of whom received in fact an off-scale dose. For v2, in both the Figure 5 caption and the main text of **On ‘tuning’**, I propose to note the important role that **fail-safe upper bounds** on both *absolute dose* and *dose multipliers* will play in reducing the risk of overshooting in a practical trial.

 A larger point about DTAT requires clarification in v2. The technical connections with *recursive filtering* and *optimal control*, as drawn in the paper, serve much the same **notional** purpose as choosing docetaxel as the basis for simulation. The above discussion shows the DTAT principle applicable to oncology therapeutics beyond the cytotoxics. Likewise, notwithstanding the essential heuristic role *recursive filtering* has played in DTAT’s development, it should not be thought to define DTAT. (In fact, I have at this point in my further work already abandoned the linear Kalman filter approach indicated in the last sentence of v1 **Abstract**, in favor of full-information methods.) What **does** define DTAT’s essential contribution to Phase I oncology study design is that it yields a **new abstraction** (the *DTA* with its *tuning parameters*) capable of *embodying knowledge objectively* [4], to supersede a **fallacious abstraction** (‘the’ MTD) that almost completely lacks this capability. I must make this point explicit in v2, emphasizing that the role of the technical connections I’ve drawn is *to illustrate* the **(DTA+tuning)** abstraction which constitutes DTAT’s essential contribution. I will also try to modify the title somehow to underscore this point. I will however retain a withering treatment of ‘the’ MTD, an anti-precision idea whose time has passed. In support of that view, I will in the v2 **Discussion** or **Introduction** briefly discuss ‘the’ MTD specifically as a *fallacy of misplaced concreteness* [5]. The purpose of my strong title is **to provoke long-overdue critical thought and discourse.** Should any clinical trial methodologist leap now to the defense of ‘the’ MTD, I will most heartily welcome that challenge.

 References

1. Weber JS, Yang JC, Atkins MB, Disis ML. Toxicities of Immunotherapy for the Practitioner. *JCO*. 2015;33(18):2092-2099. doi:10.1200/JCO.2014.60.0379.
2. Postel-Vinay S, Gomez-Roca C, Molife LR, et al. Phase I trials of molecularly targeted agents: should we pay more attention to late toxicities? *J Clin Oncol*. 2011;29(13):1728-1735. doi:10.1200/JCO.2010.31.9236.
3. Molife LR, Alam S, Olmos D, et al. Defining the risk of toxicity in phase I oncology trials of novel molecularly targeted agents: a single centre experience. *Ann Oncol*. 2012;23(8):1968-1973. doi:10.1093/annonc/mds030.
4. Popper KR. *Objective Knowledge: An Evolutionary Approach*. Rev. ed. Oxford [Eng.] : New York: Clarendon Press ; Oxford University Press; 1979.
5. Whitehead AN. *Science and the Modern World: Lowell Lectures, 1925*. New York: The Free Press; 1997.

# Peer review: Referee response for version 1

**DOI:** 10.5256/f1000research.11448.r20080

**Published:** 2017-03-14

**Authors:** Matthew E. Nielsen

**Affiliations:** 1 Departments of Urology, Epidemiology, and Health Policy & Management, University of North Carolina at Chapel Hill, Chapel Hill, NC, USA

The author presents a provocative modeling-based demonstration of an innovative alternative to the traditional one-size-fits-all maximally tolerated dose concept. The concept of individualized pharmacokinetic-based precision dosing is intuitively appealing and the analyses presented herein support potential utility from the development and application of such methods.

I have read this submission. I believe that I have an appropriate level of expertise to confirm that it is of an acceptable scientific standard.
